# Altered Regional Homogeneity with Short-term Simulated Microgravity and Its Relationship with Changed Performance in Mental Transformation

**DOI:** 10.1371/journal.pone.0064931

**Published:** 2013-06-03

**Authors:** Yang Liao, Danmin Miao, Yi Huan, Hong Yin, Yibin Xi, Xufeng Liu

**Affiliations:** 1 Department of Psychology, Fourth Military Medical University, Xi'an Shaanxi, China; 2 Department of Radiology, Xijing Hospital, Fourth Military Medical University, Xi’an Shaaxi, China; Institute of Psychology, Chinese Academy of Sciences, China

## Abstract

In order to further the insight into the explanation of changed performance in mental transformation under microgravity, we discuss the change of performance in mental transformation and its relationship with altered regional homogeneity (ReHo) in resting-state brain by using simulated weightlessness model. Twelve male subjects with age between 24 and 31 received resting-state fMRI scan and mental transformation test both in normal condition and immediately after 72 hours −6° head down tilt (HDT). A paired sample t-test was used to test the difference of behavior performance and brain activity between these two conditions. Compare with normal condition, subjects showed a changed performance in mental transformation with short term simulated microgravity and appeared to be falling. Meanwhile, decreased ReHo were found in right inferior frontal gyrus (IFG) and left inferior parietal lobule (IPL) after 72 hours −6° HDT, while increased ReHo were found in bilateral medial frontal gyrus (MFG) and left superior frontal gyrus (SFG) (*P*<0.05, corrected). Particularly, there was a significant correlation between ReHo values in left IPL and velocity index of mental transformation. Our findings indicate that gravity change may disrupt the function of right IFG and left IPL in the resting-state, among of which functional change in left IPL may contribute to changed abilities of mental transformation. In addition, the enhanced activity of the bilateral MFG and decreased activity of right IFG found in the current study maybe reflect a complementation effect on inhibitory control process.

## Introduction

Human is the subject in space exploration activities, whether his activity is normal is the key point for all sorts of space exploration tasks, thus how the space environment influence individual activities had became a generally focus among space medical and psychological researchers. It is commonly believed that individual cognitive and behavioral activities would be impaired in space environment because there are negative factors like microgravity, radiation, noise and fatigue, etc [1], [2], [3], [4]. Among those negative factors, microgravity factor is the main difference between space environment and the earth environment, so relevant researches was a hot topic in recent years and there were many significative findings [5].

While exposed to microgravity environment, astronauts always face problems such as changed body positions and need to recognize objects and other astronauts’ gestures from various perspectives [6]. Due to the perspective change make the recognition process difficult, astronauts usually need to use the mental rotation strategy (mentally rotate an internal representation of the object) to help complicating it. If one couldn’t successfully completed the mental rotation process and following recognition process, he would fail to correctly recognize the object and sign around the cabin wall as well as crewmembers’ gestures, accordingly, it would bring obstacles to activity in the cabin and communication between crewmembers. So, the mental rotation ability plays a significant role in living in space and correlated researches are of great importance. Based on this kind of demand, several previous studies had done some excellent work on observing the variation of mental rotation ability under microgravity environment [7], [8], [9].

When Matsakis et al. initially discussed this issue in 1993, they suggested that the rate of mental rotation was increased in weightlessness as compared to pre-flight values by observing two astronauts’ performance in a mental rotation task [9]. Afterwards, Leone et al. came to a different conclusion in 1995. They declared that no deterioration of the mental rotation abilities of subjects occurring in space as compared to their own abilities prior to and after the flight [8]. In the above two studies, the applied stimuli are both nonsensical three-dimension objects, the results have limited value in practical application. While the stimuli are meaningful body parts, which commonly defined by the term mental transformation instead, it will be more close to practical application. However, it is a pity that research on mental transformation ability under microgravity has been seldom reported. As we have known, Grabherr et al. once made effort to discuss this issue by the method of parabolic flight. In their study, they found that task that requires the mental transformation of one’s own body or body parts became more difficult during microgravity [7].

Indeed, compared to previous mental rotation researches, mental transformation research has its merit in higher application value. Nevertheless, the amount of reported literature is quite a few and the recent study is tracking studies which just simply use behavioral test to discuss subjects’ mental transformation ability change. In this mean, the result is not very credible and just a phenomenological discuss in a relatively primitive stage. Meanwhile, parabolic flight model is usually applied to simulate the transient change induced by microgravity [10]. During the parabolic flight, there exists frequent gravity change, which may bring in mix factors such as acute stress. What’s more, due to the experiment environment is particular and advanced brain science facilities is nearly unable to fit in this environment [11], the internal neural mechanism of mental transformation ability change has not yet been clarified.

Fortunately, as a generally accepted technology in studies of microgravity among space science researchers [12], −6° head down tilt (HDT) bed rest could stimulate the microgravity effect of redistribution of individual body fluid toward head in a relative longer duration than parabolic flight, thus it makes it is possible to explore the effect of microgravity on individual cognitive and behavioral activities on earth. In addition, with the development of the experimental techniques of cognitive neuroscience, the application of functional magnetic resonance imaging (fMRI) technology in psychological research makes the exploration of the neural mechanism of the cognitive and behavioral processes possible [13], [14]. Especially, the resting-state fMRI technology could overcome the limitations of inconsistent results caused by different experimental designs and support more stable and effective evidence in the exploration of individual brain activities [15]. Moreover, previous studies have propose that resting-state fMRI data can be used as an alternative to task fMRI data, which is of great importance when task fMRI data are not easily or accurately acquired [16]. Thus, it had been accepted as an effective technology in the research of internal neural mechanism of individual cognitive and behavioral activities. For this technology, regional homogeneity (ReHo) is a widely used new method in resting-state fMRI research [17]. In ReHo analysis, Kendall's coefficient concordance (with value from 0 to 1) is used to calculate the coherence of time series of a given voxel with those of its nearest neighbors (26 voxels), thus we could learn the synchronization of the brain activities in a quantitative way. Our previous ALFF study has indicated that a gravity change-induced redistribution of body fluid may disrupt the baseline of local brain activity. But it should be noted that brain is a complex organ, it more emphasis on the interaction between neurons. Thus to clarify the brain activity change with short term microgravity from the perspective of functional connectivity (Such as ReHo, functional connectivity, brain network) is very meaningful. In addition, we could verify the result of the previous study in a different method by carry out the current study.

In the current study, to address the question how and why astronauts’ mental transformation ability change in microgravity, we combined the resting-state fMRI technology and −6° head down tilt bed rest technology together to find the difference of ReHo as well as behavior reaction change in a mental transformation test before and after 72 hours of −6° HDT bed rest. The results may support some evidences and references to further the insight into the explanation of changed performance in mental transformation under microgravity and rich the knowledge about protection of astronauts’ brain function.

## Materials and Methods

### 2.1. Subjects

Sixteen health subjects were recruited in the present study; four subjects were excluded from the analysis because of excessive head motion (more than 2 mm or 2 degree in any axis). Totally, data of 12 subjects met the above mentioned criteria. Due to most of the astronauts are males, the subjects we selected are normal adult males. The valid subjects’ mean age is 26.4, range from 24 to 31. They were all right-handed as measured by Handedness Questionnaire [18]. Subjects reported no history of neurological injury, genetic mental disorders and substance abuse. With high resolution T1- and T2-weighted MRI examination, no subjects were found to have significant pathological change in brain. The study was approved by the Ethical Committee of the Fourth Military Medical University and all participants provided their written informed consent before the experiment.

### 2.2. Design

A pair comparison of subjects’ resting-state brain activities between normal condition and simulated microgravity condition were applied in the present study. The collected data of normal condition was subjects’ resting-state brain activities under normal seating posture condition. The collected data after simulated weightlessness condition was subjects’ resting-state brain activities after 72 hours −6° HDT bed rest. During the HDT bed rest, adequate water and foods were supplied to subjects and subjects’ head were forbidden to get out of bed in order to keep the redistribution of individual body fluid toward head. In order to avoid the fMRI scanning order of two conditions becoming an interference factor, the order of the scanning of the two conditions were balanced. Half of the subjects’ data of simulated microgravity condition were collected ten days after the data collection of normal condition while the others data collection order reversed. Moreover, in order to avoid the interference of biological rhythms, data collections were all carried out in the same time period in a day.

### 2.3. Behavioral Experiment

#### 2.3.1. Experimental paradigm

**Figure 1:**
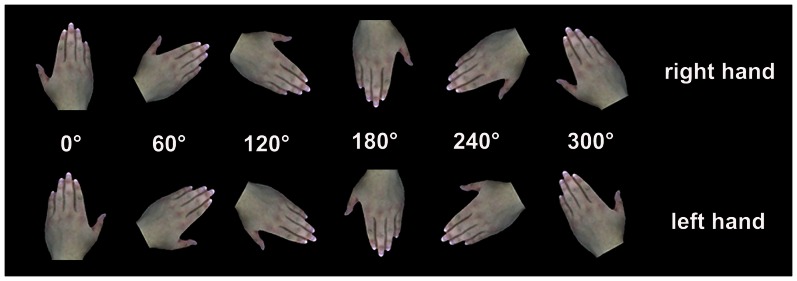
Stimulus used in the mental transformation test.

Subjects were asked to complete the mental transformation test on a computer. The stimulus of hand pictures are shown in Fig. 1. At the beginning of each trial, a cross was presented in the center of the screen for a random duration (the duration ranging from 200 ms to 500 ms) by a commercial visual software (Stim2.0; Neuroscan Inc., USA). Initially the cross disappeared; the stimulus began to present until the subject complete the judgment by pressing the mouse button. Subjects’ task was to mental transformation the stimulus into upright position and judge whether the stimulus is right hand or not. There were six kind of orientation (0°, 60°120°, 180°, 240°, 300°) for both right and left hand pictures. Except for 0° and 180° stimulus repeated 60 times during the whole test, the other types stimulus all repeated 30 times. The sequences of trials were random by the computer.

#### 2.3.2. Analysis of behavioral data

Those trials which were rightly responded were accepted for the further analysis. The reaction time (RT) between stimulus onset and response was averaged across clockwise and counterclockwise orientation angles. For previous studies have demonstrated that there was no statistical differences between counter- and clockwise stimulus orientations in the same angle [19], we merged them into four rotation conditions into the following linear regression analysis. The four conditions are: (i) stimuli not rotated (0°), (ii) 60° rotation (included stimuli rotated 60°/300°), (iii) 120° rotation (included stimuli rotated 120°/240°) and (iv) 180° rotation (included stimuli rotated 180°). Linear regression analysis on raw RTs as a function of the angle of rotation (angle of rotation ranging from 0° to 180°) was carried out. These linear regressions gave us the slope of the regression line. While rotated the same orientations, the higher slope means the subject needs more time to complicate the mental transformation process. Then the inverse of the slope corresponds to the velocity of the mental transformation, that is, if a subject has a higher slope in the regression analysis, he ought to have a slower velocity of the mental transformation.

#### 2.3.3. Statistic

To explore the velocity of the mental transformation differences between the normal condition and simulated microgravity condition, a pair-sample t-test was performed.

### 2.4. Image Acquisition and Analysis

#### 2.4.1

Data acquisitions. Additionally, all subjects’ image data were collected by an experienced radiologist with a Siemens 3.0T nuclear magnetic resonance equipment in the radiology department of the Xijing Hospital of Fourth Military Medical University. Subjects were told to lay on the scanning platform flatly and place the head to the assigned location cozily. Sponge earplugs were used to plug the ear hole in order to minimize the interference of noises and some foam padding were used to restrict head motion. A standard 16-channels head coil was used to collect the data. The subjects were told to try to keep the head motionless, to keep calm with eyes closed and to not think of anything in particular. (In addition, the data of functional images after 72 hours stimulated weightlessness was collected in head down tilt position. We placed a special foam pad on the platform of the scanner, then the subjects could keep head tilt down position during data collection.) The scanning sequence and parameters were using the following settings: 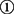 anatomical images for location were T1-weighted images which used spin echo method (SE). TR = 500 ms, TE = 15 ms, flip angle (FA) = 90°, field of view (FOV) = 220 mm×220 mm, matrix = 256×144, slice thickness = 4 mm with no gap. 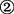 the high definition three dimensional T1-weighted images of the whole brain were used MPRAG sequence. TR = 2530 ms, TE = 3.39 ms, inversion time = 1100 ms, FA = 7°, FOV = 256 mm×256 mm, matrix = 256×192, slice thickness = 1.33 mm with no gap, 128 slices. 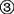 an echo planner imaging sequence was used to acquire functional images, TR = 2000 ms, TE = 30 ms, FA = 90°, FOV = 200 mm×200 mm, matrix = 64×64, slice thickness = 3 mm, gap = 0.6 mm, 33 slices, this part of scan contained 240 time points and lasted 8 minutes.

#### 2.4.2. Data preprocessing

Data Processing Assistant for Resting-State fMRI (DPARSF, http://www.restfmri.net/forum/DPARSF) V2.0 was used in data preprocessing [20]. The preprocessing steps were as follows: First, the data format of functional images was transform from DICOM to NFTI. Second, because of the subjects need some time to adjust, the first ten time points were discarded and 230 time points were left. Third, the left functional images were slice-time corrected, and aligned to the first image of each session for motion correction. Four subjects were excluded from the analysis because of excessive head motion (more than 2 mm or 2 degree in any axis). Fourth, the data was then spatially normalized with the Montreal Neurological Institute (MNI) template (resampling voxel size = 3×3×3 mm^3^)in the Resting-State fMRI Data Analysis Toolkit (REST, www.restfmri.net) v1.0 [21]. Fifth, Linear trend was removed and then the fMRI data were temporally band-pass filtered (0.01 ≤ f ≤ 0.08 Hz) to reduce the low-frequency drift and physiological high-frequency respiratory and cardiac noise. Then head motion, white matter and CSF signals were regressing out before ReHo computation as previous studies suggested [22], [23], [24].

#### 2.4.3. ReHo analysis

Kendall’s coefficient of concordance (KCC) was used to measure regional homogeneity or similarity of the ranked time series of a given voxel with its nearest 26 neighbor voxels in a voxel-wise way [25], [26]:
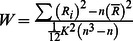
where *W* is the KCC among given voxels, ranging from 0 to 1; *R_i_* is the sum rank of the *i*th time point; 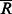 = ((n+1)*K*)/2 is the mean of the *R_i_*; *K* is the number of time series within a measured cluster (27, one given voxel plus the number of its neighbors); and *n* is the number of ranks. DPARSF was used to calculate the KCC value of the time series of a given voxel with those of its nearest neighbors (26 voxels) in a voxel-wise analysis. The normalized ReHo map was got by divide the mean KCC value of all voxels from the KCC value of each voxel of the whole brain. Then the ReHo maps were spatially smoothed (4 mm FWHM Gaussian kernel).

#### 2.4.4. Statistics

To explore the ReHo differences between the normal condition and simulated microgravity condition, a pair-sample t-test was performed on the normalized ReHo maps. The resultant statistical map was set at a combined threshold of *P*<0.005 and a minimum cluster size of 832 mm^3^ (13 voxels), corresponding to a corrected threshold of *P*<0.05, as determined by AlphaSim. For presentation purposes, the statistical maps were transformed to Talairach coordinates [27] and superimposed on the higher-resolution anatomical template available in REST. The difference areas found in paired-sample t-test was saved as masks separately in MRIcron (http://www.nitrc.org/projects/mricron). Then they were used to exacting out the mean ReHo values of these areas in Matlab7.1 (http://www.mathworks.cn/products/matlab/). Finally, these mean ReHo values was recorded into SPSS14.0 (http://www-01.ibm.com/software/cn/analytics/spss/products/statistics/) to calculate their correlations with velocity index of mental transformation.

## Results

### 3.1. Behavioral Result in Mental Transformation Test

**Figure 2:**
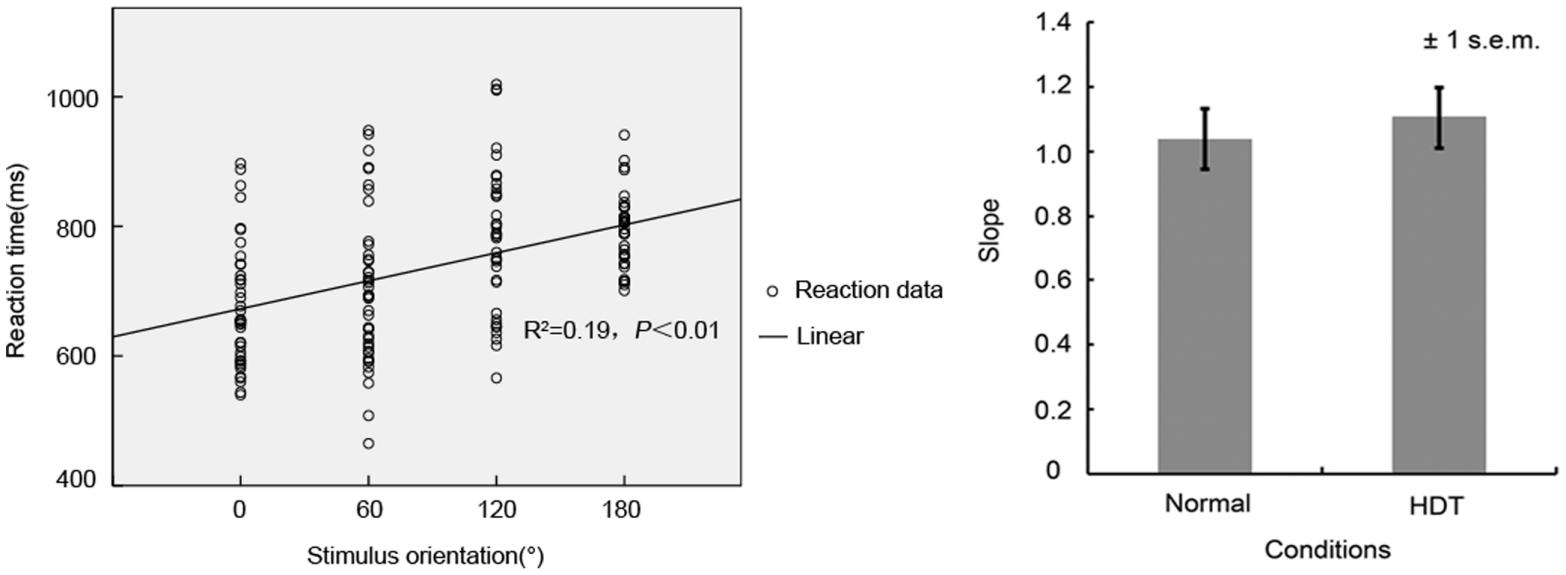
Behavioral results of mental transform test. Left: Lineal regression of reaction time and stimulus orientation in mental transform test (Subject 1 in normal condition as the example). Right: Velocity index of mental transformation in normal condition and simulated microgravity condition.

The mean accuracy in behavioral test under normal condition is 0.976 (SEM = 0.006), the mean accuracy in behavioral test after HDT is 0.973 (SEM = 0.004). There is no significant difference in mean accuracy in behavioral test between these two conditions (*P* = 0.736). The reaction times in mental transformation test are shown in Fig. 2. Regression analysis suggest that there are significant linear trend between reaction time and stimulus orientation across all subjects (*P*<0.05). Thus the slopes of the regression lines are valid. Compared to normal condition, subjects showed increased slop of mental transformation with short term simulated microgravity which may indicate declined mental transformation ability in microgravity. But from a statistical point of view, this difference was not significant at 0.05 level.

### 3.2. ReHo Results

**Figure 3:**
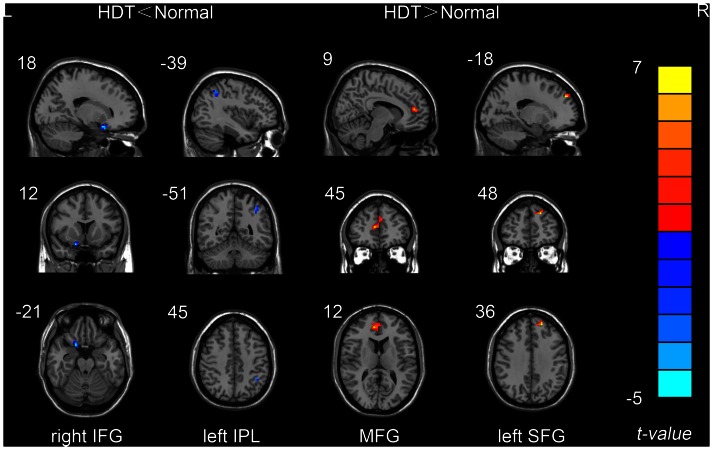
Brain areas with significant ReHo differences between simulated microgravity condition and normal condition (A combined threshold of *P*<0.005 and a minimum cluster size of 832 mm^3^ (13 voxels), corresponding to a corrected threshold of *P*<0.05, as determined by AlphaSim).

ReHo results between the normal condition and simulated microgravity condition are shown in Fig. 3 and Tab. 1. Compared to normal condition, decreased ReHo was found in right inferior frontal gyrus (IFG) and left inferior parietal lobule (IPL) in simulated microgravity condition. In addition, increased ReHo were found in bilateral medial frontal gyrus (MFG) and left superior frontal gyrus (SFG) in simulated microgravity condition.

**Table 1:** Brain areas with significant ReHo differences between stimulated microgravity condition and normal condition.

Brain regions	Brodmann	Voxels size	Talairach coordinates			Peak-*t*
	area	(mm^3^)	X	Y	Z	value
Stimulated microgravity condition< Normal condition						
right IFG	47	1216	17	11	−14	−5.41
left IPL	40	896	−34	−53	40	−4.61
Stimulated microgravity condition >Normal condition						
MFG	10	4480	9	39	19	7.21
left SFG	9	832	−15	40	41	6.74

(A combined threshold of *P*<0.005 and a minimum cluster size of 832 mm^3^ (13 voxels), corresponding to a corrected threshold of *P*<0.05, as determined by AlphaSim).

### 3.3. The Correlations between ReHo Values and Velocity Index of Mental Transformation

**Figure 4:**
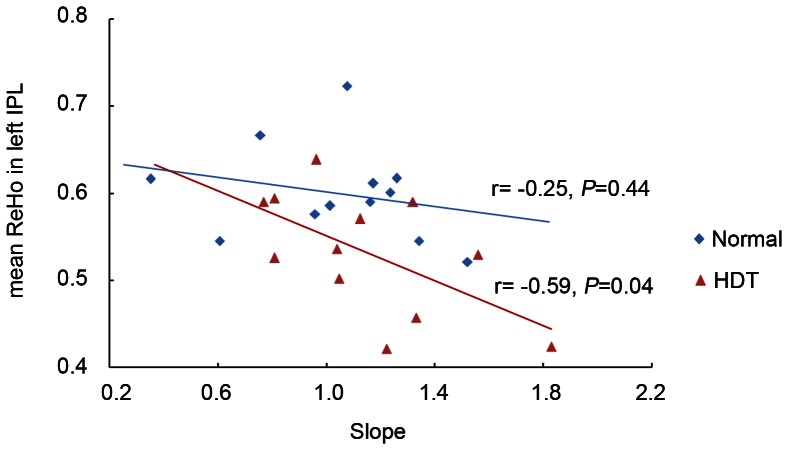
Pearson correlation analyses examining the relation between mean ReHo values in left parietal lobule and velocity index of mental transformation.

In clusters for which significant group differences had been detected, we also tested for possible correlations between ReHo and velocity index of mental transformation. Finally, there was only a significant correlation between mean ReHo values in left inferior parietal lobule and velocity index of mental transformation in simulated microgravity condition(r = − 0.59, *P*<0.05; Fig. 4).

## Discussion

The major finding of present study is the decreased ReHo in left parietal lobule with short term simulated microgravity and its significant correlation with velocity index of mental transformation. As a good quantitative indicator for reflecting the localized coherence of brain activity, ReHo could indicate the change of neural activities in certain brain areas [17], [28], [29], [30], [31], [32], [33], [34]. Thus, the ReHo changes found in the current study may reflect corresponding function changes, to some extent; it could help to clarify the internal neural mechanisms of mental transformation ability change under microgravity. Additionally, the declined trend of mental transformation ability and changed ReHo values in right inferior frontal gyrus, bilateral medial frontal gyrus and left superior frontal gyrus found with short term simulated microgravity are worthy discussed.

At behavioral level, a declined trend of mental transformation ability was found with short term simulated microgravity. This was accordance with the previous study carried out by Grabherr et al. with parabolic flight model [7]. They owed this result to the disturbed vestibular function induced by the missing of gravity information input. In contrast, the model to simulate microgravity used in the present study is different; the gravity information input still exists in HDT bed rest [12]. This difference may induce weaker damage to subjects in the present study than in Grabherr’s and could partly explain the insignificant between group differences in behavioral data found in the present study. Though it is, the result could support the idea of declined mental transformation ability with microgravity presented by Grabherr et al. in some extent. However, it is worth noting that with the gravity information input still existed in present study, there may be other contributors that contribute to the mental transformation ability change. The behavioral data in the present study is not enough to clarify this issue; we should look for some evidence from the image data.

As the result found in the present study, mean ReHo vaule in left inferior parietal lobule has a significant correlation with the velocity index of mental transformation. Accordingly, the declined function of left inferior parietal lobule revealed by the decreased ReHo in the left inferior parietal lobule with short term microgravity may play a significant role in mental transformation ability change. Amount of previous studies have provided credible evidence to this inference. Initially, Kosslyn et al. found the bilateral inferior parietal lobule activation in the mental rotation of hand pictures by positron emission tomography [35]. Later, an fMRI study carried out by Vingerhoets et al. similarly found the bilateral parietal areas activated during the mental transformation task [36]. Similar finding was reported again by Overney in 2005, they suggested that left parietal regions were strongly activated during mental transformation [37]. Thus, left inferior parietal lobule is with no doubt play an important role in the processing of mental representation (especially the mental representation of body parts, such as hand). The decreased ReHo found in this area with short term microgravity in the present study is ought to reflect a dysfunction there, which may in turn disturb the mental transformation process. Accordingly, the changed mental transformation ability found in the present study could be explained reasonably. Nevertheless, based on the data of the current study, we couldn’t conclude the reason of the dysfunction in left inferior parietal lobule yet. A possible reason is that the redistribution of individual body fluid toward head induced by simulated microgravity may affect the neuron metabolism in the left inferior parietal lobule, but this assumption lack direct evidence to support. An alternative assumption is that the dysfunction of left inferior parietal lobule is caused by the declined vestibular function. There are some indirect evidences to verify it in the previous studies. Several studies have reported the vestibular dysfunction in HDT bed rest [7]. Moreover, Overney et al. ever suggested that the parietal areas need to receive the input information input vestibular during the mental transformation process [37]. While vestibular function declined during simulated microgravity, the vestibular-cortical interactions would be disturbed in sequent, which may accordingly disturb the neural activity in left inferior parietal lobule (which plays an important role in mental transformation process). For now, the latter assumption seems to be more credible, but it is still need to be clarified in the future. Even though, the dysfunction in left inferior lobule with short term simulated microgravity and its relationship with changed mental transformation ability found in the present study is valuable, it will further the insight of brain activity change in microgravity and promote the development of corresponding countermeasures.

Moreover, it is worth paid attention to that declined function in right inferior frontal gyrus and enhanced function in bilateral medial frontal gyrus were also found in the present study. Previous studies [38], [39], [40], [41] have demonstrated that individual frontal cortical functioning was inhibited under microgravity and in turn impair several psychological processes such as inhibitory control etc. But enhanced frontal areas activity in microgravity had been seldom reported. As far as we know, amount of previous findings had suggested that right inferior frontal gyrus and medial frontal gyrus play critical roles in inhibitory control process [42], [43], [44], [45], [46], [47], [48], [49], [50]. Therefore, the enhanced activity of the bilateral medial frontal gyrus found in the current study maybe reflect a compensatory mechanism to the declined inhibitory control process. But due to there is no behavioral data for reference, whether this compensatory mechanism is stronger enough to cancel out the negative effect caused by the abnormal activity of the right inferior frontal gyrus is not clear yet. The relation of these two areas and their impact on inhibitory control is still a problem that worthy be addressed.

In addition, hyperfunction in left superior frontal gyrus after short term simulated microgravity was found in the present study. In previous studies, superior frontal gyrus has been related to multiple cognitive processes, such as task-switching, expression identification and complex motor control [51], [52], [53], [54], [55]l. Due to the lack of corresponding behavioral data, we could hardly clarify the exact role of hyperfunction in left superior frontal gyrus found in the present study; it needs to be addressed in the future studies.

The findings of the present finding are encouraging, but there still exist some limitation that need to be addressed. Firstly, because of technical limitations, the sampling rate (TR = 2 s) used in the data acquisition is low; therefore, simultaneously recording of cardiac rates is impossible [56], [57]. Although we could not eliminate the cardiac rate interference from our data, our findings may seem to be reliable after multiple corrections in some extent. Secondly, those findings of present study are gained from the simulated experiment on earth and there are some limitations when they are extended to microgravity environment. These findings are still needed to be tested in real microgravity environment in the near future.

### Conclusion

In conclusion, gravity change may disrupt the function of right inferior frontal gyrus and left inferior parietal lobule in the resting-state, among of which functional change in left inferior parietal lobule may contribute to changed abilities of mental transformation. In addition, the enhanced activity of the bilateral medial frontal gyrus and decreased activity of right inferior frontal gyrus found in the current study maybe reflect a complementation effect on inhibitory control process. However, these results are gained from the simulated experiment on earth and there are some limitations when they are extended to microgravity environment. These findings are still needed to be tested in real microgravity environment in the near future.
